# Wearable sensor and smartphone assisted vestibular physical therapy for multiple sclerosis: usability and outcomes

**DOI:** 10.3389/fresc.2024.1406926

**Published:** 2024-07-10

**Authors:** D. Meldrum, H. Kearney, S. Hutchinson, S. McCarthy, G. Quinn

**Affiliations:** ^1^ School of Medicine, Trinity College Dublin, Dublin, Ireland; ^2^ MS Unit, Department of Neurology, St. James’s Hospital, Dublin, Ireland; ^3^ Physiotherapy Department, St. James’s Hospital, Dublin, Ireland

**Keywords:** vestibular rehabilitation, remote monitoring, multiple sclerosis, dizziness, disequilibrium

## Abstract

**Introduction:**

Vertigo, dizziness, gaze instability and disequilibrium are highly prevalent in people with MS (PwMS) and head movement induced dizziness is commonly reported. Vestibular physical therapy (VPT) is a specialised, non-invasive and effective therapy for these problems but usually involves travel for the person to a specialist center with both personal and carbon costs. The use of wearable sensors to track head movement and smartphone applications to deliver and track programs has potential to improve VPT in MS.

**Methods:**

This study investigated the usability and effects of a commercially available digital VPT system (wearable head sensor, smartphone app and clinician software) to deliver VPT to PwMS. A pre/post treatment design was employed and the primary outcome was the System Usability Scale (SUS). Other patient reported outcomes were the Service User Acceptability Questionnaire (SUTAQ), the Patient Enablement Instrument (PEI) and the Dizziness Handicap Inventory (DHI). Physical outcomes measurements included Mini-BESTest (MB), Modified Dynamic Gait Index (mDGI), Gait Speed (GS), Dynamic Visual Acuity (DVA) and head kinematics and symptoms during exercise.

**Results:**

Sixteen PwMS (14 female), mean age 44(±14) years were recruited to the study and twelve completed VPT. Mean adherence to exercise, measured digitally was 60% (±18.4). SUS scores were high at 81 (±14) and SUTAQ scores also demonstrated high levels of satisfaction and acceptability of the system. Statistically significant improvements in MB (mean change 2.25; *p* = 0.004), mDGI (median change 1.00; *p* = 0.008), DVA (median change −1.00; *p* = 0.004) were found. Head frequencies significantly improved with concurrent decreased intensity of dizziness during head movements (mean change across 4 gaze stabilization exercises was 23 beats per minute; *p* < 0.05). Non-significant improvements were seen in DHI (*p* = 0.07) and GS (*p* = 0.15). 64.5% of follow up visits were conducted remotely (video or phone), facilitated by the system.

**Discussion:**

This study had two main outcomes and benefits for PwMS. Firstly, we showed that the system used was both acceptable and could be used by PwMS. Secondly, we demonstrated an improvement in a range of dizziness, balance and gait metrics with remotely delivered care. This system has the potential to positively impact on MS physiotherapy service provision with the potential to deliver effective remote care.

## Introduction

Multiple Sclerosis (MS) is a progressive neurodegenerative disease affecting 2.9 million individuals worldwide (1). As an autoimmune disorder, MS results in demyelination and plaque formation throughout the central nervous system. As a consequence, the cerebellum, brainstem and dorsal root entry zone of the 8th cranial nerve are common areas for plaque formation and this can be a significant factor in disequilibrium experienced by people with MS (PwMS) (2).

Numerous studies of vestibular function in MS have shown abnormalities in vestibular evoked potentials, electronystanography, static posturography (3, 4) and dynamic visual acuity (5). Furthermore, worse vestibular function is associated with greater disability (6). Vestibular dysfunction results in vertigo, dizziness, disequilibrium, and gait impairment and these are common and disabling symptoms of MS resulting in functional limitations, loss of independence, falls and an overall decreased quality of life (7–9).

Vestibular physical therapy (VPT), a specialized form of physical therapy that targets vestibular dysfunction is increasingly being employed for PwMS. In this population, VPT improves balance, quality of life and fatigue and reduces dizziness (10–13). A recent systematic review of (*n* = 7) randomized trials concluded VPT to be a safe and effective intervention in MS but acknowledged a limited evidence base (14). In most studies, VPT is delivered “face to face” in clinics for an initial assessment and for follow up visits but the treatment outcomes are thought to be dependent on a home exercise program prescribed between visits. There is currently no system for monitoring adherence and technique remotely (14).

Current evidence for VPT in peripheral vestibular dysfunction supports exercising in short bouts up to five times a day (15). With the frequency that the exercise program needs to be performed, there are unsurprisingly a number of barriers. These include, but are not limited to, motivation, lack of feedback and guidance, as well as symptom provocation (16).

An apparent paradox presents itself for the PwMS being treated, they attend VPT to improve dizziness but the exercises prescribed will generally provoke symptoms. Given the prevalance of dizziness with head movement, which is estimated to affect as many as three quarters of PwMS, an effective treatment regime is critical to improve quality of life (17).

Improving head movement and dynamic visual acuity are core aims of VPT, and the exercises most commonly prescribed are gaze stabilization exercises (15, 18, 19). These exercises involve the individual focusing on a stationary target when moving their head in either the pitch or yaw plane and are known as vestibular ocular reflex times one exercises (VORx1). They are performed with the target presented at near (N), and far (F) distances, and the frequency and duration of the exercise, as well as the position the individual exercises in (e.g., sitting, standing) are progressed as tolerated (19).

Technological advances, such as wearable technologies linked to electronic records present opportunities for addressing the problems of symptom control and improved adherence to prescribed exercise programs. Web based VPT has been shown to be effective in chronic dizziness (20, 21) but does not address the problem of accurately measuring adherence or providing biofeedback during exercise. Sensors such as accelerometers, and gyroscopes allow human physiological signals to be encoded and recorded, allowing health professionals to measure patient exercise performance and adherence in ways that were not possible previously. These forms of technology provide patients with accurate feedback of their performance which may motivate and improve rehabilitation outcomes.

There is emerging evidence that smartphone and/or wearable sensor assisted medical care for telehealth is feasible and warrants further investigation (22, 23). Loyd et al. (23) recently investigated the use of head worn inertial measurement units (IMUs) during VPT for PwMS and vestibular dysfunction. The IMUs were worn at 3 exercise sessions over a 6-week intervention period but only during clinic visits. Initial support for their ability to detect improvements in head kinematics during gaze stabilization exercises was found (23). These advances have the potential to create novel approaches to remote feedback during treatment as well as outcome metrics.

The COVID-19 pandemic highlighted the potential benefit of remotely delivered care. Prior to this as little as 4.5% of therapists reported using telehealth in VPT programs (18) but this has increased to 38% mid-pandemic (24). This provided benefits for many, in particular, PwMS embraced use of telehealth with 69.8% reporting the experience of remote care as either good or very good (25).

However, despite the clear acceptability of remotely delivered physiotherapy and prevalence of dizziness in MS, no study to date has yet investigated the provision of VPT in MS using a wearable sensor and smart phone app in the home. Therefore, the aim of this study was to address this research gap by investigating the usability of a bespoke digital vestibular rehabilitation application. The objectives of the study were threefold; firstly, to quantify the usability of the application and sensor. Secondly, to measure patient reported outcomes after VPT delivered with the system, and finally, to quantify physical outcomes.

## Materials and methods

**Figure 1:**
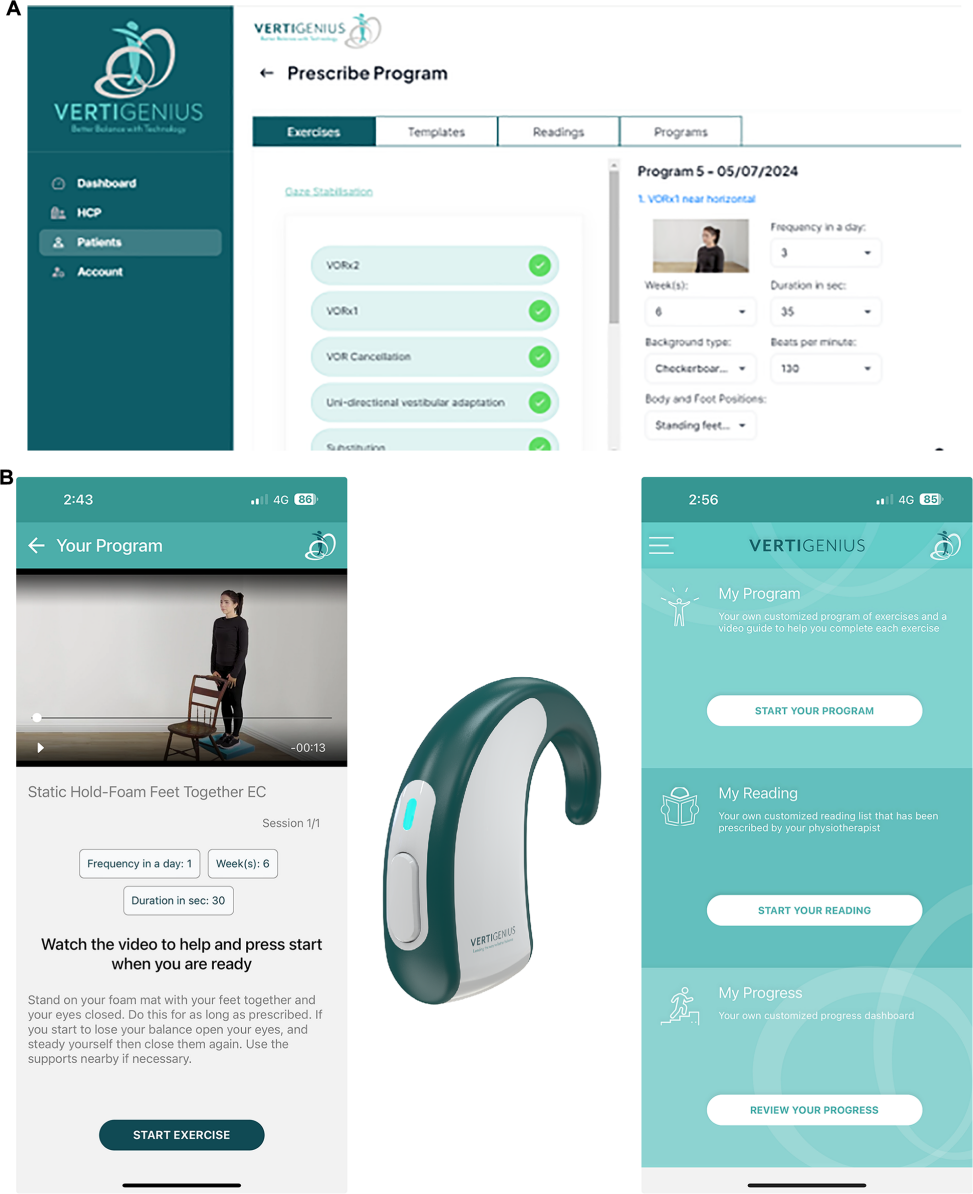
The Vertigenius™ system. Participants downloaded the application to their smartphone, which connected to the sensor which was worn behind the ear. (**A**) The clinician used the clinician software to prescribe, adjust and monitor exercise. (**B**) The head sensor information collected during exercise at home was relayed to the clinician portal and presented graphically to show whether the gaze stability exercise was performed, performed at the correct frequency in beats per minute (BPM) and dizziness symptoms before and after the exercise (not shown).

This was a usability study using a pre-treatment-post treatment design with an aim to investigate the use of a bespoke VPT system with wearable head sensor (Vertigenius™) in the delivery of VPT to PwMS. The digital VPT system consisted of a wearable head sensor, smartphone app and clinician software (Figure 1). We measured the primary study outcome using the System Usability Scale Score (SUS). We included a range of appropriate secondary outcomes measures as follows;

1. Service User Technology Acceptability Questionnaire (SUTAQ) (26)
2. Patient Enablement Instrument (PEI) (27, 28).
3. Changes in frequency of head movement and evoked dizziness during four gaze stabilization exercises (VORx1 near and far and in vertical and horizontal planes).
4. Dizziness Handicap Inventory Score (29).
5. Dynamic Visual Acuity (30, 31).
6. Modified Clinical Test of the Sensory Interaction on Balance (32).
7. Gait Speed (33).
8. Modified Dynamic Gait Index (34).
9. Mini-BESTest (35).
10. Adherence to the application and sensor (automatically measured by the sensor and system).
11. Daily numerical rating scale (NRS) score of dizziness, imbalance, nausea, anxiety, and oscillopsia (participant inputted via the app).
12. EQ5D5l Health Thermometer (36).

Data collection took place in the MS clinic and the physiotherapy department of a large university teaching hospital, where we identified and approved PwMS for recruitment to this study. Ethical approval was obtained from the hospital's Medical Research Ethics Committee. The study aimed to recruit 12–15 participants which is considered an adequate sample size for the primary outcome, the SUS (37, 38).

Inclusion criteria for the study were as follows: (i) diagnosis of MS (39) (ii) independently mobile with or without an aid, (iii) willing to use a smartphone/sensor health application, (iv) age >18 years, and (v) active dizziness, vertigo, or imbalance confirmed via subjective (Self report yes or no) or objective measures (balance abnormalites detected by the treating PT on the Mini-BESTest, see below). Exclusion criteria were as follows: (i) fluctuating vestibular disease (active Meniere's disease, migrainous vertigo), active benign paroxysmal positional vertigo, or other medical conditions in the acute phase (e.g., orthopaedic injury), (ii) pregnancy, (iii) MS relapse or change in disease modifying therapies in the past three months. Relapse was measured by clinical features e.g., new symptoms or change in symptoms and by MRI findings e.g., new lesions or increasing size of current lesions.

### Procedure

At baseline participants underwent the following assessments recommended by either the Vestibular Evidence Database to Guide Effectiveness (VEDGE) or the Multiple Sclerosis Task Force of the American Physical Therapy Association (40).

1. Ten meter (m) walking test (33). Participants walked at their preferred speed along a 10 m track in the clinic. The instruction: “please walk to the end of the room at your normal pace” was used. A lead in of 1 m was given and the participant continued to walk past the 10 m mark. Gait speed (GS) was then calculated in meters per second (m/s).
2. The 4 item modified Dynamic Gait Index (mDGI). This is a validated four-task assessment of walking function: (i) gait at self-preferred speed, (ii) gait when changing speeds, (iii) gait with horizontal head turns, and (iv) gait with vertical head turns. It is scored from 0 to 12 with higher scores representing better gait function (34).
3. Mini-BESTest (MB). This is a balance assessment which measures dynamic balance, including: anticipatory transitions, postural responses, sensory orientation, and dynamic gait. The MB was selected over the Berg balance test for this MS study as it has demonstrated a lower ceiling effect in this context (35).
4. Dynamic Visual Acuity (DVA). This was measured using an ETDRS (Early treatment of diabetic retinopathy) chart. Static visual acuity (SVA) was first determined as the lowest LogMAR line at which participants could correctly identify 3 out of 5 optotypes. The therapist then assisted the participant to move their heads at 120BPM and asked them again to identify the optotypes. The line at which participants could correctly identify 3 out of 5 optotypes was then compared to SVA and the difference calculated in number of lines of LogMAR lost (30).
5. Dizziness Handicap Inventory (DHI). This is a patient reported outcome measure for those with vestibular dysfunction, and has been validated in MS (41). It consists of 25 questions and is scored on a Likert scale yielding a total score of 0–100 percent. Higher scores indicate higher burden of symptoms.
6. Usability and Enablement. At the conclusion of treatment, three questionnaires were administered to assess the usability (defined as acceptability, learnability, and ease of use) and enablement aspects of VPT delivered with the application and sensor.
  A. The System Usability Scale (SUS). This questionnaire was designed to subjectively assess usability of interface technologies. Levels of agreement with ten statements are scored using a five-point Likert scale anchored with “strongly disagree” and “strongly agree”. The SUS provides a point estimate of percentage usability. Scores of above 70 are acceptable and highly usable products score above 90. Scores below 50 indicate unacceptably low levels of usability (37, 38).
  B. The Service User Technology Acceptance Questionnaire (SUTAQ). This questionnaire was developed to quantify patient's beliefs and expectations with regard to their acceptability of a tele-health system that included “kit”, which in the case of this study was the head sensor and app. The questionnaire has 22 statements that are agreed or disagreed with on a six-point Likert Scale (ranging from strongly disagree to strongly agree). Six subscales are returned by the questionnaire measuring constructs of enhanced care, increased access, privacy and discomfort, care personnel concerns, kit as substitution and satisfaction (26).
  C. The Patient Enablement Instrument (PEI), has been used to evaluate quality of health care consultations in primary health care. It consists of six questions about change, both in patients' ability to cope with their condition, and in their understanding of their condition. It is scored on a 0–12 point scale with higher scores indicating greater enablement (27, 28).
7. Change in subjective symptoms on 0–10 numerical rating scales including change in symptoms with prescribed head frequencies during gaze stabilization exercises.
8. Percentage adherence to exercise (collected automatically by the system and duration of treatment (in weeks).
9. Care provision associated cost questionnaire. Participants filled out a questionnaire relating to cost of attending in time, distance and financial terms and were asked about falls since the previous visit.

### Intervention

After baseline measures and an initial assessment by the treating physiotherapist (GQ) were completed, an individualized treatment plan was decided and discussed with the participant. Participants were onboarded to the system using a pseudonymous code. The system consists of a clinician portal where prescription of an individualized exercise program takes place and thereafter tracks exercise adherence and symptoms by electronically sending a range of subjective questionnaires.

Participants were shown how to download the app to their smartphone. Once registered on the clinician software, the treating physiotherapist selected and electronically sent their individualized program to them and showed them how to use the application. At each subsequent clinical visit and until discharge, revised and progressed exercise programs were prescribed as appropriate. The exercises prescribed included a combination of adaptation, habituation, balance and gait exercises, as would be traditionally used in VPT but delivered through the interface of the smartphone app rather than using pen and paper or an exercise print out.

Use of the app allowed the participant to watch a professional video of each prescribed exercise prior to doing the exercise and the app provided counts and timers for exercises and an audible metronome, the frequency of which was prescribed by the therapist. Examples of videos and interface may be viewed at https://www.vertigenius.com/. The app automatically progressed the participants through their exercises and measured their subjective responses to gaze stabilization exercises (vertigo/dizziness, nausea and disequilibrium) on a numerical rating scale. The app also provided digital reminders to complete the exercises and information on progress (change in vertigo, nausea, imbalance, anxiety and oscillopsia as well as head frequency during exercise, and adherence). Educational materials specific to balance and inner ear problems and tailored to the participant could also be prescribed by the portal and presented in the app.

Each participant received a head sensor (VG01; Figure 1) for use at home for gaze stability exercises. The sensor contains an inertial measurement unit (IMU) with a dual axis gyroscope, sampling at 50 Hz to measure angular velocity of head movement (degrees/s), in both yaw and pitch axis orientation. Angular velocity is used to estimate the frequency in beats per minute (BPM) of head rotation in either axis. VG01 internally processes the angular velocity of the head movement, finding a zero crossing on the head velocity and uses this to calculate BPM and subsequently sends the corresponding BPM values directly to the mobile phone app. Real-time feedback on head movement is attained by using Bluetooth technology to stream head BPM and max velocity from the head sensor to the mobile phone app at 10 Hz. The head sensor provided real time feedback on correct frequency of the head movement in relation to the prescription. The app alerted the participant through use of a traffic light system where the target on the phone screen if the participant was moving the head too fast, was red, too slow was yellow, or at the correct frequency, was green. Every second day, at one exercise session, the participant was asked to rate their symptoms of dizziness before the exercise started and after the exercise finished. This information was digitally collated and relayed back to the clinician portal, allowing the clinician to see graphs of the percentage adherence to the gaze stability exercise, the percentage time during the exercise going too fast, too slow or at the prescribed frequency and the level of symptoms before and after the exercise.

The initial assessment and final assessment were carried out in person in the physiotherapy department but for review sessions all study participants had the option of a telehealth consult (a phone or video call) if they so desired. At each session a cost analysis questionnaire was completed which collected data on time off work for the consultation, parking and transport costs, and any other costs e.g., childcare, food etc. Participants were also asked if they had missed any time off work due to vestibular symptoms since the preceding session and if they had experienced any falls or had needed a medical review due to fall related injuries.

### Data analysis

Data relating to the participant's interaction with the application was processed by two of the researchers (DM and GQ). Descriptive statistics were used for the analysis of demographic data, of SUS, SUTAQ, PEI scores and cost questionnaires. Descriptive statistics were also used to analyze the number of programs and exercises prescribed and percentage adherence to the programs. Data were examined for normality using histograms and QQ plots. Paired *t*-tests and Mann–Whitney *U*-tests were used to investigate pre- and post-treatment scores in normally and non-normally distributed outcomes respectively (DHI, MB, GS, DVA, mDGI, NRS scores). Change in head frequencies during gaze stabilization exercises and change in dizziness symptoms during the four gaze stabilization exercises pre and post treatment were also examined using paired *t*-tests.

## Results

A total of 16 participants (14F), mean age 44 (±14) years consented to the study, twelve completed the study. Demographics and baseline characteristics are shown in Table 1. Four withdrew from the study. Reasons for withdrawal were severe fatigue (*n* = 1), nausea (*n* = 1, not related to the intervention), moved elsewhere (*n* = 1), did not adhere to treatment with no reason given (*n* = 1).

**Table 1:** Demographics and baseline characteristics.

Variable	Mean	SD	Range
Age	44.1	13.6	25–67
Disease duration (years)	11.5	9.9	0.1–32
Expanded disability status scale (EDSS)	2.5^a^	1.1^a^	0–6
Number of falls in past year	2.1	3.05	0–10
Sex Male (%)	12.5		
Female (%)	87.5		
MS subtype Relapsing remitting (%)	93.7		
Secondary progressive (%)	6.3		
Variable	Yes (%)		
Mobility aid use	25		
History of falls	56.3		
Fear of falls	37.5		
Employed (FT or PT)	56.3		
Vertigo	93.8		
Dizziness	87.5		
Oscillopsia	50		
Imbalance	87.5		
Head motion intolerance	62.5		
Headache	56.3		
Fatigue	75		
Aural symptoms (any of the 3)	62.5		
Tinnitus	56.3		
Aural fullness	25		
Deafness	25		

Demographics table for *N* = 16 that have baseline and prevalence data.

For numerical values, reported as mean and SD.

For categorical, yes/no questions, reported as percentage of yes.

^a^ Median and Interquartile range. FT, fulltime; PT, part time.

### Treatment intervention

The duration of VPT was on average 12 (±2.2) weeks (range 7–14). A mean of 5.5 (±1.2) programs were prescribed during this time with a duration of 2.2 (±0.5) weeks. The therapist prescribed 9 (±1.2) exercises per program. Overall mean adherence to the exercises prescribed was 60.1 ± 18.4% (range 28%-88%). There was no statistically significant correlation between SUS scores and overall percentage adherence (*r* = 0.32, *p* = 0.3).

At baseline, participants reported traveling a median distance of 5.9 km to the initial session (range 1–210 km) taking a median of 25 min (0–180). Half reported being unable to fulfil a family or work role due to dizziness in the past month, and five (31.3%) reported a fall in the past month. Nine of 16 (56.3%) participants were employed and four had taken time off work to attend treatment. Of the 60 subsequent clinical consultations before the final in person assessment, 48 (64.5%) were conducted via either video or tele consult. Reasons given in support of tele/video consults were preferable to a long commute, convenient, less time consuming, had no requirement for childcare, had flexibility, and were less costly. Reasons against tele/video consults were a preference for face to face and limited technology abilities. At follow up assessments, there were eight further falls reported by *n* = 4 (25%) of participants.

### Usability

**Figure 2:**
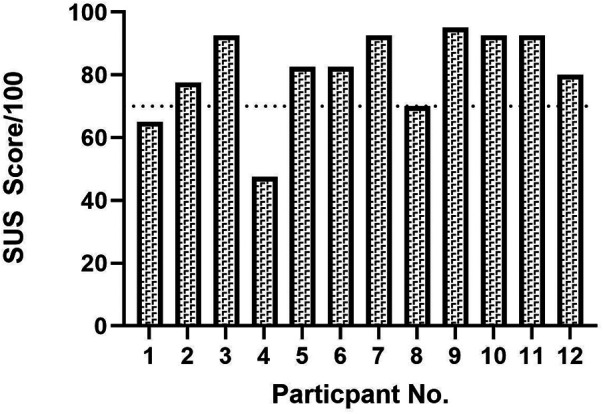
System usability scores (SUS) by participant. The SUS is scored out of 100 with higher scores representing higher usability of a given system. A cut-off of 70 (denoted by the dotted line) is the cut-off score for usability.

Mean SUS score was 81 (±14; range 47.5–95), displayed in Figure 2. On average participants agreed strongly, or very strongly, with the statements relating to finding the system easy to use, quick to learn, and confidence using it. On average, they strongly disagreed with the statement “I thought the system was unnecessarily complex”. There was less agreement with the statement “I think I would like to use the system frequently” with only 3/12 participants strongly agreeing with this statement and the remainder scoring 2/5 or 3/5 (A score of 5/5 was anchored with the statement strongly agree and 0/5 anchored with the statement strongly disagree). Only two participants scored below the accepted cut-off of 70.

### Enablement scores

Mean PEI scores were 5.8/12. The majority of participants selected “better” or “much better” when answering all statements related to enablement but approximately one third reported feeling the “same or less” with regard to the six statements in the instrument (Table 2).

**Table 2:** Results from the Patient Enablement Instrument (*n* = 12).

As a result of your visit to your PT today do you feel you are?	% Scoring same or less	% Scoring better/much better
Able to cope with life	33	67
Able to understand your illness	25	75
Able to cope with your illness	25	75
Able to keep yourself healthy	25	75
Confident about your health	33	67
Able to help yourself	33	67

### SUTAQ

**Figure 3:**
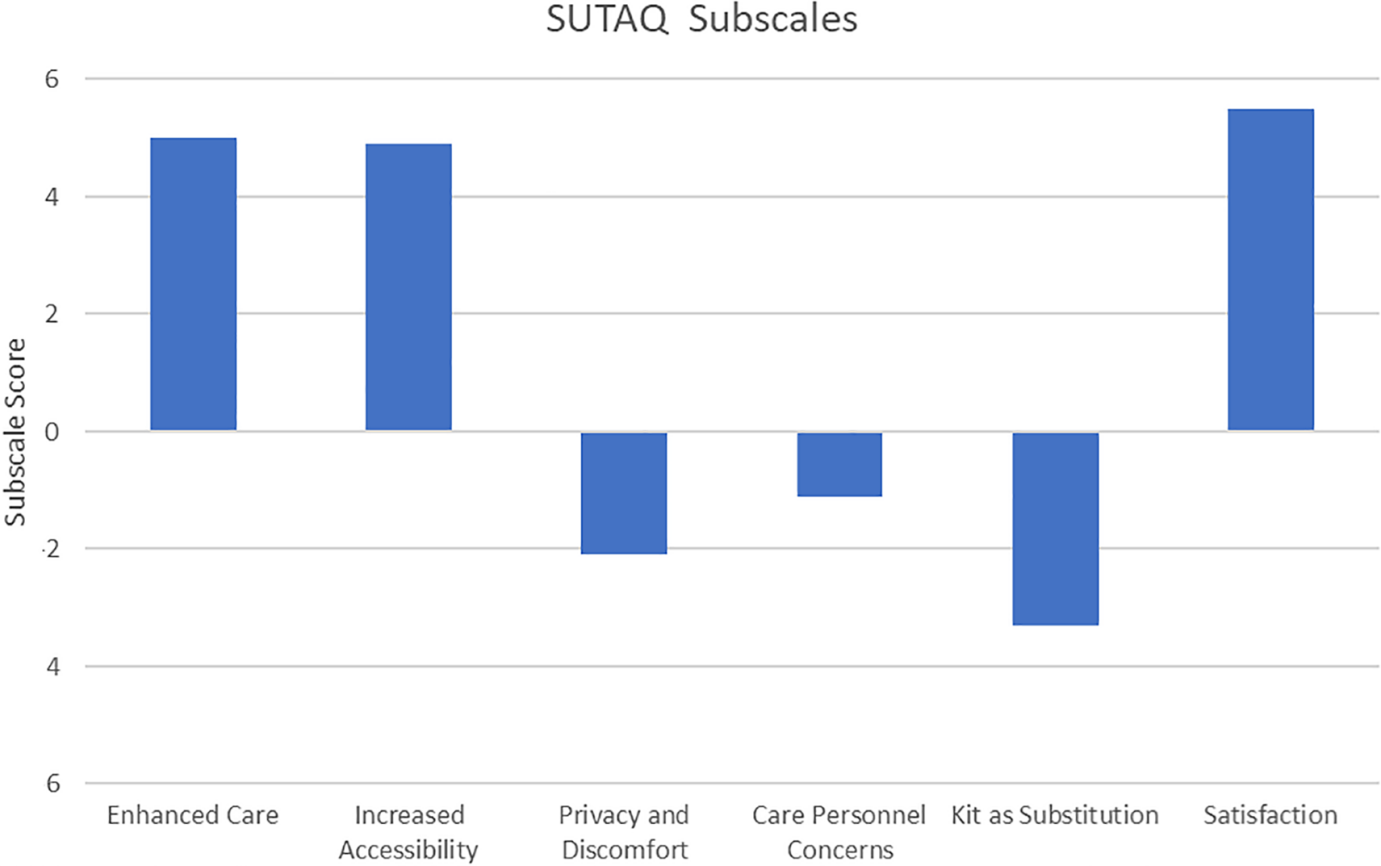
Service user acceptability technology questionnaire (SUTAQ) subscale scores. The subscales, named in the legend above had a max score of 6. The Privacy and Discomfort, Care Personnel concerns and Kit as Substitution scales are shown below the x axis as high values on these scores reflect high levels of agreement with negative aspects of the “kit”.

SUTAQ sub scale scores were calculated according to Hirani et al. (26). High average scores (out of a maximum of 6, higher indicating agreement) were evident for the scales measuring whether the participant felt the kit enhanced their care (mean score 5.0), increased their access to care (mean score 4.9) or their overall satisfaction with the kit (mean score 5.5) (Figure 3). For example, on item 1 “The kit I received has saved me time in that I did not have to visit my GP clinic or other health/social care professional as often”, 100% agreed with this statement and for item 15, “The kit can be/should be recommended to people with a similar condition to me”, 100% also agreed. Participants scored low on the privacy and discomfort scale indicating they had minimal concerns (mean score 2.1). They also scored very low on the care personnel concerns (mean score 1.1) i.e., they agreed the professionals providing the sensor and app and care were competent and continuity of their care was not affected by the system. There was ambiguity on the “kit as substitution” subscale (mean score 3.3); 67% of participants agreed with the statement that “the kit is not as suitable as regular face to face consultations with the people looking after me”.

### Physical and symptom outcomes

Statistically significant improvements were found for Dynamic Visual Acuity (median score pre-intervention of 2 lines lost vs. a post intervention score of 1 line lost, *p* = 0.004), Mini-BESTest Scores [mean score pre-intervention of 23 (± 2.8) vs. a post intervention score of 25 (±2.6), *p* = 0.004] and Modified Dynamic Gait Index scores (median score pre-intervention of 11 vs. a post intervention median score of 12 *p* = 0.008). Non statistically significant improvements (0.05 m/s) were observed for gait speed (*p* = 0.15), Dizziness Handicap Inventory scores (*p* = 0.07), Modified CTSIB scores (*p* = 0.2) and EQ5D5l Health Thermometer scores (Table 3). NRS scores for dizziness, oscillopsia, nausea and imbalance all showed statistically significant reductions (Table 3).

**Table 3:** Pre and post outcomes of physical and subjective outcome measures.

Outcome	Mean T1 (SD)	Mean T2 (SD)	Diff (SD)	*P*-value	95% CI
DHI (/100)	46 (13)	37 (17)	−8.8 (15.4)	0.07	−18.6→0.96
Gait Speed (m/s)	1.3 (0.17)	1.4 (0.13)	0.05 (0.11)	0.15	−0.02→0.12
Mini-BESTest (/28)	23 (2.8)	25 (2.6)	2.3 (2.1)	0.004	0.89→3.6
Health Thermometer (/100)	67 (17)	68 (18)	1.4 (18.0)	0.79	−10.0→12.9
NRS Dizziness (/10)	3.9 (1.9)	1.5 (1.2)	−2.4 (1.8)	0.002	−3.6→−1.1
NRS Imbalance (/10)	4.0 (1.5)	1.5 (1.2)	−2.5 (1.5)	0.0003	−3.5→−1.5
NRS Anxiety (/10)	2.8 (3.4)	0.39 (0.53)	2.4 (3.2)	0.07	−5.1→0.3
NRS Oscillopsia (/10)	3.8 (1.3)	1.5 (1.1)	−2.3 (0.8)	0.0003	−3.0→−1.5
NRS Nausea (/10)	2.5 (1.1)	0.49 (0.59)	−2.0 (0.8)	0.005	−3.0→−1.0
	Median (IQR)	Median (IQR)		*P*-value	
DVA (no. of lines lost)	2 (2, 4)	1 (1, 2)	–	0.004	–
mDGI (/12)	11 (9.3, 12)	12 (11, 12)	–	0.008	–
mCTSIB (/120 s)	108 (95, 120)	116 (105, 120)	–	0.2	–

DHI, dizziness handicap inventory; m/s, metres per second; NRS, numerical rating scale; DVA, dynamic visual acuity; mDGI, modified dynamic gait index; mCTSIB, modified clinical test of the sensory interaction on balance; IQR, inter quartile range; SD, standard deviation; Diff, difference, CI, confidence interval.

### Head kinematics

**Figure 4:**
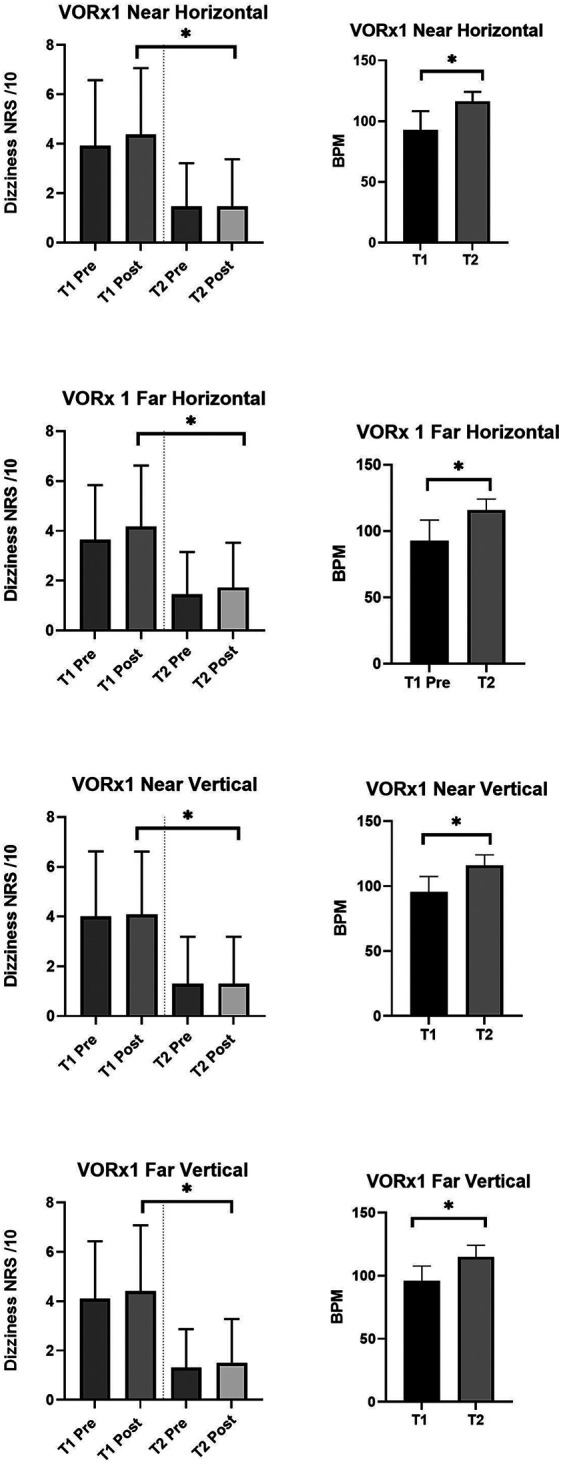
Change in dizziness numerical rating scores (out of 10) before and after the four gaze stability exercises both at time 1 and time 2. Time 1 is the first program prescribed and Time 2 is the last program prescribed. On the left It can be seen that for all exercises, the symptoms were higher at T1 than at T2. At both T1 and T2 pre and post exercise scores did not increase significantly. On the right, graphs showing the change in prescribed frequency of head movements at T1 and T2. Participants significantly increased the frequency at which they performed the exercises from T1 to T2 with concurrent decrease in symptoms. *p < 0.05. VORx1 Vestibular ocular reflex times one exercise, NRS Numerical Rating Scale, T1 Time one, T2 Time 2, BPM Beats per minute (frequency at which the participant was performing the exercise with real time feedback of performance via the head sensor and app).

Eleven of the 12 participants used the head sensor during their gaze stabilization exercises. One participant was unable to connect to their smartphone (an older version) but continued to use the app for exercise instruction but without the sensor feedback. This resulted in no sensor data relating to head kinematics being available for this participant. All participants were prescribed four gaze stabilization exercises (VORx1 at near and far distances and in the pitch (vertical) and yaw (horizontal) planes), except one participant who was not prescribed Vertical VORx1. Figure 4 shows subjectively rated dizziness before and after performing individual gaze stabilization exercises in the initial and final programs. Overall, symptoms were not exacerbated excessively with the exercises and, over time, symptoms of dizziness showed statistically significant decreases for all four exercises. Concurrently, prescribed head frequencies increased significantly for all four gaze stabilization exercises indicating that overall participants were moving their heads at faster frequencies with less dizziness at the end of treatment.

### Adverse effects

No treatment related adverse effects were reported during the study. Of eight falls reported during the study by four participants, none occurred during performance of the prescribed exercises.

## Discussion

This study addressed an unmet research gap by delivering remote VPT with real-time feedback of exercise performance for PwMS. The outcomes included improvements in symptomatology, documented by a range of both subjective and objective metrics. We also found high levels of acceptability and usability of the technology in people with this chronic neurological disease that has an extremely high prevalence of dizziness. This has the potential to impact MS care by facilitating remote delivery of specialized VPT and importantly addressing barriers to adherence to the prescribed exercise where exacerbation of symptomatology is frequently encountered.

Rehabilitation is a cornerstone of management of MS, and tele-rehabilitation has previously been shown to positively affect quality of life (42). Next generation systems incorporating virtual reality and sensors, such as those used successfully in this present study have potential to augment tele-rehabilitation improving access to treatment, outcomes, and increasing understanding of dosage and effects of different exercises and approaches for dizziness in MS (23, 43, 44).

The results from this study showed that participants found the system highly usable, based on the results from two usability questionnaires (SUS and SUTAQ). SUS scores above 70 are deemed acceptable, and a mean score of 81 obtained in this present study was encouraging in this regard although two participants scored below the threshold of 70. These scores are in agreement with a previous study on the system in peripheral vestibular disorders (45). The SUTAQ more comprehensively evaluated constructs of how the “kit” was perceived and participants scored highly on the constructs of enhanced care, increased accessibility and satisfaction. The observation that 64.5% of follow up consultations were performed remotely supports the SUTAQ score; both participants and the therapist involved reported saving time as a result of the use of the system. Disagreement was evident amongst participants in the perception of whether the kit could be used as a replacement of care, with 42% of participants disagreeing that “the kit can be a replacement for my regular health or social care”. This suggests that a hybrid approach to VPT in PwMS might be the most valued, but requires further study as the field of tele-rehabilitation is relatively new and equivocal results have been obtained (46). Van Vugt et al. (20) used web based VPT with or without the addition of two home visits by a therapist and compared the groups to a usual medical care group in a chronic dizziness population. No differences were found between the two intervention groups and both improved more than the usual medical care groups. Qualitative interviews supported the home visits as valued by both patients and therapists despite adding some cost to the intervention (47).

The head sensor had several functions in the delivery of VPT. Firstly, it gave real time feedback to participants during gaze stability exercises. These exercises have a good evidence base in vestibular disorders (15) but are known to increase symptoms and patients often report difficulty with performing them correctly, meeting the right head frequency and motivating themselves to exercise (16). Secondly, the head sensor tracked head frequency and coupled with the participant inputting subjective dizziness scores before and after exercises (once a day, every second day) provided the therapist with accurate real-time information on exercise performance and effects. It can be challenging to prescribe optimal head frequency and therapists currently use symptoms to guide prescription. This approach lacks oversight of what is happening with home exercises and therapists rely on what the patient reports and the head sensor allowed remote therapeutic monitoring and possibly aided and enhanced the proper performance of exercises at home. Clinically significant increases in the ability to move the head at progressively higher frequencies and with less dizziness were objectively measured which is encouraging. The head sensor also digitally measured exercise adherence, a metric which is acknowledged as being central to advancing knowledge of exercise dosage and effect in VPT (15). A mean exercise adherence of 60.1% was recorded which was not ideal, but similar to previous studies of VPT (48, 49). In VPT, poorer outcomes are associated with reduced adherence (50) and adherence is poorly measured in studies of exercise interventions in MS and VPT (15, 51). Percentage adherence did not correlate with SUS scores suggesting that the technology was not the reason for low adherence. Furthermore, on closer inspection, the participant with the lowest adherence (28%) in the present study had a low burden of symptoms at inception and improved quickly, which may have impacted their adherence. Future studies using wearable sensors coupled with digital exercises interventions such as the system employed in this study will be able to accurately determine adherence to exercise, whether better outcomes are possible with increased adherence and which exercises are most beneficial.

### Physical outcomes

The study was not powered to assess efficacy and it is acknowledged that a randomized controlled trial is necessary for this. However, statistically significant improvements were found for balance, DVA and the mDGI. A 3 line or more loss of visual acuity is considered abnormal in DVA testing but most healthy subjects will not drop more than one line (31). All participants at baseline had a DVA loss of 2 lines or more and nine improved DVA post treatment. This suggests that the function of the vestibular ocular reflex was improved after treatment and supported by a statistically significant reduction in subjectively reported oscillopsia and lends further support to the use of gaze stabilisation exercises in PwMS. The mean increase in Mini-BESTest scores was 2.3. This did not reach published MDC scores for MS of between 3.5 and 4.7 (52), but reached the 10% MDC improvement calculated by Mitchell et al (53). mDGI scores also increased significantly, indicating better gait function. Gait speed increased by 0.05 m/s but was not statistically significant.

### Subjective outcomes

One of the most commonly used subjective measures of dizziness is the Dizziness Handicap Inventory (18, 54). DHI scores reduced by 8.8 which was non-significant but similar to a report by Loyd et al (12) who reported a reduction of 8–9 when face to face VPT was provided to a sample of PwMS. It was less than that found by Hebert et al (11) in a study of VPT in PwMS who found a clinically significant reduction of 18.7. The differences may be explained by treatment duration, VPT in the latter had a time frame of 14 weeks, and/or disease duration, which was 6 years, as opposed to 11 years in the current study. However, in the present study, dizziness measured with numerical rating scales showed significant decreases. The DHI is an overall measure with constructs of physical, emotional and functional effects of dizziness which may account for the disparity.

### Limitations of the study

We included only PwMS who were able to mobilise independently with or without a gait aid, this limits the generalizability of the study to the PwMS population who may have a greater range of disability levels and disease progression. The study was powered to assess usability but was underpowered for effectiveness. Some outcomes may have not reached significance due to low numbers producing a type II error. In addition, there was no control group and a future randomized controlled trial is necessary to evaluate efficacy, particularly of the sensor and digital approach compared to conventional VPT. We did not cost an episode of care and more robust economic data is necessary before the cost-effectiveness of this digital approach can be quantified. The effects on fatigue were not formally assessed and 75% of participants reported fatigue at baseline with one dropout due to severe fatigue. A previous study found that VPT significantly improved fatigue (11) and in future studies, a daily digital NRS measure of fatigue could be incorporated to the system. We also did not include a measure of cognition which may have influenced results. Finally, the intervention duration may not have been long enough and long-term follow up of the improvements observed was not conducted.

## Conclusion

This study has demonstrated high usability of a wearable head sensor combined with a digital application for VPT in PwMS. The system was well tolerated and accepted with no adverse events and reductions in dizziness at increasing head frequencies were observed with concurrent improvements in balance and gait.

## Data Availability

The raw data supporting the conclusions of this article will be made available by the authors, without undue reservation.
